# Characterization of Fed Cattle Mobility during the COVID-19 Pandemic

**DOI:** 10.3390/ani11061749

**Published:** 2021-06-11

**Authors:** Sage Mijares, Michelle Calvo-Lorenzo, Nick Betts, Lacey Alexander, Lily N. Edwards-Callaway

**Affiliations:** 1 College of Veterinary Medicine and Biomedical Sciences, Colorado State University, 1601 Campus Delivery, Fort Collins, CO 80523, USA; mijaress@rams.colostate.edu; 2 Elanco Animal Health, 2500 Innovation Way, Greenfield, IN 46140, USA; MICHELLE.CALVO_LORENZO@elancoah.com (M.C.-L.); NICK.BETTS@elancoah.com (N.B.); 3 Cargill Protein Headquarters, 825 E Douglas Ave, Wichita, KS 67202, USA; Lacey_Alexander@cargill.com; 4 Department of Animal Sciences, Colorado State University, 1171 Campus Delivery, Fort Collins, CO 80523, USA

**Keywords:** cattle, COVID-19, days on feed, feedlot, mobility, welfare

## Abstract

**Simple Summary:**

Cattle mobility is an important animal welfare outcome measured by beef producers and meat processors. Factors that negatively impact fed cattle mobility include high environmental temperatures, heavy body weights, handling practices during loading and unloading, and longer transport times. As the United States cattle industry recovered from closures and/or reduced capacity at slaughter plants that occurred as a consequence of the COVID-19 pandemic, there was concern that cattle mobility challenges would intensify due to the increased prevalence of some of the previously identified risk factors, particularly as summer months approached. The aim of this study was to characterize cattle mobility during the COVID-19 pandemic recovery period at a slaughter facility located in the Central Plains United States. Although mobility challenges increased at the study facility from July through October 2020 as compared with historical benchmarking databases, the prevalence of cattle with significant impairment did not increase and remained low. Mobility scores were impacted by average weight, temperature humidity index, distance hauled, sex, and days on feed. Although mobility challenges increased during this time period, collaborative efforts across the supply chain were effective at managing mobility conditions important to cattle welfare during the marketing and slaughter process.

**Abstract:**

The COVID-19 pandemic had significant consequences on cattle slaughter capacity in the United States. Although industry stakeholders implemented strategies to minimize cattle welfare impacts of increased weights, days on feed (DOF), and increasing temperatures, there were concerns that mobility challenges would be observed at slaughter facilities. The objectives of this study were to characterize mobility in fed cattle during this recovery period and to identify factors impacting mobility. A total of 158 groups of cattle (15,388 animals) from one slaughter facility were included in the study. A 4-point mobility scoring system was used to assess cattle mobility. Cattle at the facility with normal mobility scores were reduced from the historical average of 96.19% to 74.55%. No increase in highly elevated mobility scores was observed. Mobility was impacted by weight, temperature humidity index (THI), distance hauled, sex, and DOF, with results differing by mobility category. Weather was a key contributor to mobility challenges; the relative risk of observing an elevated mobility score was 45.76% greater when the THI changed from No Stress to Mild Stress. Despite the challenges that the industry faced during this period, efforts to minimize negative effects on cattle welfare by enhanced focus on low-stress handling were effective.

## 1. Introduction

COVID-19 was declared a pandemic by the World Health Organization on 11 March 2020 [1]. By late March 2020, meat processing plants in the United States began to shut down or reduce capacity in order to control the number of COVID-19 infections among processing plant workers. Throughout April, May, and June, processing plants intermittently closed or slowed production in order to control COVID-19 outbreaks [2]. Closing processing plants and slowing production lines within plants allowed for greater social distancing between employees [3], but negatively impacted meat processing plant output and created risk for downstream impacts on animal welfare. Beef processing plants were particularly impacted during this time. For the 8 weeks following 5 April 2020, cattle slaughter averaged 22% lower than the same time period in 2019 [4]; the total number of cattle slaughtered in the United States in May 2020 was 23 percent less than the same time the previous year [5]. Overall, cattle slaughter numbers were drastically impacted, with estimates ranging from 25% to 40% loss in production capacity and slaughter numbers for beef cattle nationally [3,4,6,7].

As beef processing plant capacity was reduced, live cattle were retained longer in feedlots [8]. Cattle marketings, or cattle shipped out of feedlots to slaughter, were down 28% in May 2020 compared to May 2019 [9]. The decrease in marketings resulted in a shift towards more days on feed (DOF) for cattle in feedlots. The retention of cattle on feed for more days forced producers to change diets with the goal of slowing growth so animals would still be an appropriate market weight to process even after extended DOF. Strategies to achieve lower rates of gain included changing diets to include more roughage, limiting feeding to 90% of the previous ration, and reducing the use of growth implants [8]. Nationally, the average live weight of cattle at slaughter in May of 2020 was up 23 kg from the previous year, to 620 kg [5].

The number of DOF required before cattle are ready to slaughter varies based on a variety of factors, such as sex, starting weight, slaughter weight, and feed costs [10]. The number of cattle on feed for more than 180 days (COF > 180 days) is used as an indicator for how many animals are being marketed; as COF > 180 days increases, fewer cattle are being marketed and more animals are being retained on feed in the feedlot [11]. To provide a perspective on the backlog of cattle inventory due to reduced slaughter capacity, on 1 July 2020 feedlots with capacity of 1000 or more head had 252% greater COF > 180 days compared to July 2019 [11], highlighting that market disruptions were persistent several months after plant restrictions were lifted.

As a consequence of these market disruptions due to the pandemic, animal welfare issues across livestock and poultry industries became apparent. With the inability to move animals off of the production facilities to the slaughter facilities, there was a concern for overcrowding and associated repercussions on-farm such as increased aggression, limited feed access, increased mortality, and poor mobility [12]. The pig and poultry industries were impacted more significantly due to faster growth rates, more intensive housing systems, and quicker production cycles. Producers tried to implement strategies to reduce growth such as altering diets, removing growth promoters, reducing feed availability, and altering the environment to reduce feed intake [8,13,14]. Despite exhaustive efforts, some pig and poultry producers had to depopulate some of their herd in order to manage the backlog of animals that were unable to be marketed. Although cattle producers were able to avoid these extreme measures, there were concerns regarding the potential welfare impacts that longer DOF and increased live weights would have on cattle welfare including increased mobility challenges, mortality, transportation conditions, and difficulty with animal handling.

One of the welfare concerns, particularly as summer weather approached, was an increase in the incidence of elevated cattle mobility scores, i.e., an increase in abnormal mobility. Mobility is an important animal welfare and production issue, with the beef industry placing a heightened focus on mobility issues in finished cattle over the past several years [15]. As an example, mobility scores are now included in the National Cattlemen’s Beef Association (NCBA) Beef Quality Assurance (BQA) Feedyard audit [16]. Cattle mobility is a multifactorial issue with some identified risk factors for mobility challenges including high temperatures [17], heat stress [18], heavier body weights [15], handling practices during loading and unloading [15], transport duration [15,17], and transport conditions [15]. While the impact of DOF had not been consistently quantified in past benchmarking efforts, it is hypothesized that longer DOF could also be a factor in causing mobility challenges. The circumstances and timing of the COVID-19 pandemic led to an increase in some mobility risk factors including greater cattle body weight, more DOF, and hot summer weather.

The objective of this study was to characterize mobility in a sub-population of feedlot cattle at a federally-inspected commercial slaughter plant during the COVID-19 recovery period, i.e., when the long-fed backlog of cattle were being slaughtered, and identify risk factors impacting mobility with particular focus on haul distance, weather, cattle weight, DOF, and sex/breed class as critical factors.

## 2. Materials and Methods

This study received an exemption from the Colorado State University Animal Care and Use Committee (#1501) as all procedures were non-invasive and merely observational.

A total of 16,262 cattle were observed in this study. Data were collected at a commercial United States Department of Agriculture inspected beef slaughter plant located in the Central Plains region of the United States from July through October 2020 on 54 observation days. This plant was selected based on their willingness to participate in this study. The period was selected to capture the hotter summer months. The participating facility operated in two 8 h shifts slaughtering approximately 5000 head of cattle per day. All data were collected ante mortem in the livestock holding pens. The holding pens were not covered and the flooring in the pens and alleyways was stamped concrete.

Mobility was scored on all study animals using the North American Meat Institute (NAMI) 4-point mobility scoring system [19] (Table 1). One scorer collected all mobility data throughout the entire study period. The scorer was a plant employee. Training of the scorer occurred remotely via exchange of emails, conference calls, sharing of videos, and correlation tests with two mobility scoring experts; experts participated in the original development of the mobility scoring system and had extensive experience scoring mobility in feedlot cattle. Training and correlation testing were deemed complete when the scorer achieved 100% agreement with experts using multiple training and reliability test videos from cattle footage obtained at commercial slaughter plants. After this accuracy level was achieved, data collection began.

The tracking unit of cattle ownership at the plant is the lot number, which is applied to a unique group of cattle coming from the same origin. Specific lots were selected by the scorer with the intention of sampling across different times of day, shifts, and types of cattle. The scorer observed cattle from various locations on the catwalk over the holding pens. The specific location was selected to obtain the optimal view for mobility scoring depending on the location of the target lot. Cattle were observed as a group as they were moved from a holding pen into a drive alley; some lots were viewed as they were exiting the holding pen and others were observed as they were moved down the drive alley. For each mobility score category, the number of cattle within each mobility category were tallied as cattle moved past the observer; each individual animal was scored. The number of cattle in each mobility category were summed and percentages were calculated by dividing by the total number of cattle within the lot. In addition to mobility scores, the following information was collected for each lot: sex class (i.e., steer or heifer), number of trucks in the lot, livestock trailer weights (which would be used to calculate average live weight for the lot), breed type (i.e., Holstein and non-Holstein), and date. Average live weight was determined by calculating the difference between full and empty livestock trailer weights for each load within the lot and then dividing this sum by the total number of cattle on all trailers within the lot. For each lot, producer location (i.e., zip code), country of origin (i.e., Canada and Mexico) and DOF were obtained by requesting this information from the in-plant cattle purchasing team who communicated directly with the feedlots of origin. Temperature and humidity were recorded at the beginning of each scoring session using an online platform (AccuWeather, Inc., State College, PA, USA). AccuWeather, Inc. reports hourly weather data from the nearest weather station which in the case of this study was located approximately 3 miles from the slaughter plant. Data collection sessions lasted one hour or less and therefore authors felt this weather data was representative of the targeted lot scoring time. Detailed information on transport conditions and time at the plant prior to scoring were not collected in this study due to logistics and complexity of this additional data collection.

An estimate of distance traveled to the plant was determined using Google Maps (Google LLC, Mountain View, CA, USA) to calculate an estimated distance from the plant zip code to the producer zip code. If multiple options were provided by Google Maps, the shortest route was selected and the provided distance between zip codes was recorded. The authors recognize that this distance is an estimate and actual travel distances may have varied slightly due to selected route and specific location within the zip codes utilized. The distance traveled was then grouped into three categories for analysis: < 97 km, between 97 and 193 km, and ≥ 193 km. These categories were selected due to the desire to have a minimum of 10 unique zip codes represented within each distance category. The temperature humidity index (THI) was estimated using the recorded temperature and humidity values in the following equation: 0.8 × T + H × (T − 14.4) + 46.4 where T is temperature in Celsius and H is the percentage of relative humidity [20,21]. THI is used extensively in studies looking at the impact of heat stress in cattle as a measure of heat load [22,23,24]. Based on the calculated THI, lots were categorized into associated stress categories indicating heat load risk: No Stress = THI < 72; Mild Stress = 72 ≤ THI < 79; Severe Stress = 79 ≤ THI < 89; Very Severe Stress = 89 ≤ THI < 99; and Dead Cows ≥ 99 [20,25]. There were no lots within the Very Severe Stress and Dead Cows categories.

Data from two proprietary, confidential industry benchmarking databases launched by Elanco Knowledge Solutions at Elanco Animal Health (Greenfield, IN, USA) were summarized to provide the reader with some additional industry benchmarking information on lot level characteristics relevant to the present study’s results for insight purposes. The databases were filtered so that only data from the same time period, plant, and breed type were included in summary information. For the purposes of this paper, only historical data from August through October from the years 2016 through 2019 for both databases were included. Elanco’s Cattle Mobility Assessment Program has collected fed cattle mobility data at 15 different packing plants and on more than 12M head of cattle scored individually by trained third-party evaluators using the NAMI Mobility Scoring System [19]. Elanco’s Benchmark Program captures feedyard production and health and carcass data on over 8M head of feedyard marketings annually. 

### Statistical Analysis

All data were entered into a spreadsheet (Microsoft Excel, Microsoft Corporation, Redmond, WA, USA). There were 2 lots that were considered split lots by the facility but because all characteristics (e.g., distance traveled, weather parameters) were the same for the 2 segments, these were considered 1 lot for analysis. Only native beef lots (Mexican and Holstein lots were excluded) were included in the statistical analysis. Three lots were missing observations for DOF; the average DOF of the dataset were used for these lots.

All analyses were conducted using logistic regression with JMP Pro version 15.1.0 (SAS Institute Inc., Cary, NC, USA). Explanatory variables of sex class, DOF, THI category, distance traveled category, average weight, and shift were investigated for the % of mobility scores ≥ 2 and ≥ 3. Binomial and Poisson distributional assumptions were evaluated and the Poisson model was selected for both models (mobility scores ≥ 2 and ≥ 3) as it had the lowest overdispersion. The log of lot head count was used as an offset parameter to appropriately weight the Poisson models. Explanatory variables were removed from the models in a stepwise manner based upon the highest *p*-value until all remaining variables had *p*-values < 0.05. Explanatory variables were deemed significant to the models with *p*-values < 0.05. The modeled relative risks associated with significant regression model components impacting the prevalence of non-normal mobility scores were also calculated.

## 3. Results

A total of 158 lots (98 from A shift and 60 from B shift), representing 15,388 cattle, were included in the final analyses. The average lot size was 97 ± 48 cattle (Mean ± SD), ranging from 29 to 278 cattle per lot. Table 2 provides a description of important lot level characteristics that were considered as potential risk factors associated with cattle mobility and/or utilized to construct factor categories used in analyses. Days on feed for cattle delivered to the facility was 206 ± 58.7 days (Mean ± SD) during the sample period (Table 3). Relative to industry benchmarking databases, the DOF during this COVID recovery time was numerically higher than what had been reported for both the Central Plains region (defined as Kansas and the southeastern part of Colorado) in which the study facility was located and all United States feedyard participants (primarily including TX, KS, NE, CO, IA, ID, WA, OR, and OK) during equivalent months for 4 years prior. On average, the DOF reported in this study was approximately 1.3 × greater than DOF reported in the Central Plains region between 2016 and 2019.

Table 4 includes mobility scores and average live weights from the current study sample. Additionally, historical data from the Elanco Cattle Mobility Assessment database are included. Average live weights across all databases were highly similar to those in the current study, indicating that despite a substantial increase in DOF, average live weights did not change when compared to previous year averages. However, there were some numerical differences between the mobility scores in the current study as compared with both facility and industry data in previous years. In the current study, the average percentage of cattle per lot with a mobility score of 1 was numerically lower than data collected at the facility during the same months over the previous 4 years (August through October, 2016 through 2019; 74.55% and 96.19%, respectively). Conversely, the average percentage of cattle per lot with a mobility score of 2 was numerically greater in the present study versus data collected at the facility during the previous 4 years (24.3% and 2.41%, respectively). This difference although not statistically compared was substantial; the current study reported a 10x increase in the frequency of cattle with a mobility score of 2. Historically, the study facility has a greater percentage of 1s and a lower percentage of 2s as compared to aggregate industry data (1s: 96.19% and 89.32%, respectively; 2s: 2.41% and 9.54%, respectively). It should be noted that while the frequencies of mobility scores of 1 and 2 changed during the current study time period, frequencies of mobility scores 3 and 4 remained relatively consistent.

**Figure 1:**
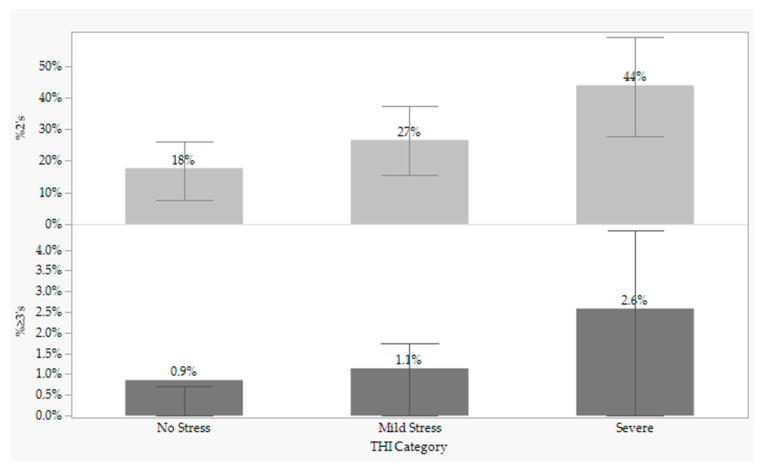
Prevalence of mobility scores utilizing the NAMI mobility scoring system [19] by temperature humidity index (THI) stress categories (*N* = 158 lots representing 15,388 head of cattle). Mobility scores were defined as: 1 = normal, walks easily, no apparent lameness, no change in gait; 2 = exhibits minor stiffness, shortness of stride, slight limp, keeps up with normal cattle; 3 = exhibits obvious stiffness, difficulty taking steps, obvious limp, obvious discomfort, lags behind normal cattle; and 4 = extremely reluctant to move even when encouraged by a handler; statue-like. THI stress categories were defined as follows: No Stress = THI < 72; Mild Stress = 72 ≤ THI < 79; Severe Stress = 79 ≤ THI < 89; Very Severe Stress = 89 ≤ THI < 98; and Dead Cows = > 99 [20]. There were no lots within the Very Severe Stress and Dead Cows categories. Error bars represent the interquartile range for each category.

The frequency of cattle exhibiting a mobility score ≥ 2 (Table 5) was impacted by: average lot weight (*p* = 0.0020), THI Category (*p* < 0.0001), Distance Category (*p* = 0.0007), Sex Class (*p* = 0.0101) and DOF (*p* = 0.0003). The frequency of cattle exhibiting a mobility score ≥ 3 was impacted by: THI Category (*p* = 0.0114), and Distance Category (*p* = 0.0117). The relationship between THI and the percentage of 2 and 3 mobility scores (Figure 1) indicated that cattle that were scored when the THI indicated Severe Stress had an almost 2.5-times greater percentage of mobility scores ≥ 2 than during No Stress (44% and 18%, respectively). A similar trend was seen with the percentage of cattle with a mobility score ≥ 3 when comparing Severe and No Stress environments (2.6% and 0.9%, respectively).

The relative risk of different THI categories (Table 6) clearly demonstrates the substantial increase in mobility challenges associated with changes in the environment. For example, the risk associated with having a mobility score ≥ 3 increases 263.58% when in a Severe Stress environment as compared with a Mild Stress environment. Similarly, comparisons between different distances traveled indicate increased relative risk associated with greater distances, particularly for mobility scores ≥ 3. The relative risk for scoring 2 or greater on the mobility scale increases as DOF and weight increase. Additionally, the relative risk for having a mobility score ≥ 2 increased 43.86% for heifers as compared to steers.

## 4. Discussion

There are many risk factors associated with mobility challenges in cattle including high temperatures [17], heat stress [18], handling practices during loading and unloading [15], transport duration [15,17], and transport conditions [15]. The Elanco Mobility Database has shown many of these same factors to be associated with elevated mobility scores including: sex, breed, season, temperature and humidity at the plant, amount of time between arrival truck weighing (occurring at animal arrival) and final truck weighing (occurring after animals have been unloaded), transport distance, and lairage duration (unpublished data). The objective of this study was to focus on key factors relevant to the COVID-19 recovery period, as well as those previously identified as significant factors influencing mobility in feedlot cattle. As the cattle industry recovered from closures and/or reduced capacity at slaughter plants that occurred as a consequence of the COVID-19 pandemic, there was concern that processors would see an increase in cattle mobility challenges due to the increased prevalence of some of the previously identified risk factors. As processors worked through the backlog of cattle inventory, producers were required to keep cattle in feedlots for a longer period of time thus increasing DOF while trying to minimize weight gain. Although not supported by published data, there was concern that cattle would have to be transported increased distances to facilities that had slaughter availability. Additionally, this recovery time occurred during the summer months and thus there was an increased risk of high temperatures and extreme heat events which had the potential to exacerbate cattle mobility challenges. Due to all these characteristics of the COVID-19 recovery period, this study aimed to gain more knowledge on fed cattle mobility at slaughter and explore the impact of key risk factors on mobility.

Mobility has been included as an outcome in studies exploring the physiological and behavioral responses of cattle to various management practices, such as shade provision, inclusion of beta-agonists in the diet, and animal handling techniques [26,27,28]. Additionally, mobility has been included as a parameter in the National Cattlemen’s Beef Association’s (NCBA) National Beef Quality Audit (NBQA) observational benchmarking studies [16,18]. The NBQA included mobility scoring for the first time in the 2016 audit using the NAMI mobility scale and reported that 96.8% of the cattle observed had a normal mobility score [16]. The NBQA included a large sample of fed steers and heifers (total cattle = 8051) over a time period that spanned many seasons (March through November). Lee et al. [18] quantified cattle mobility in an observational study and reported 2.69% 2s within the sample population, similar to Eastwood et al. [16]. The prevalence of cattle with mobility scores ≥ 2 in the current study was greater than those reported in the aforementioned studies; the percentage of 2s in the present study (24.3%) was almost 8x greater than that reported in both Eastwood et al. [16] and Lee et al. [18]. It should be noted that in previous years outside of the 2020 COVID-recovery period but during similar months, the average percentage of 2s at the current study facility was 2.41%, in line with values reported in the previously discussed studies. Comparing prevalence of mobility challenges across studies is challenging and should be done with caution as there are many risk factors associated with increased mobility scores that are not always consistent between studies, such as weather, cattle size, and distance traveled.

Other studies have explored the change in cattle mobility throughout the marketing process and have reported mobility scores more aligned with what was found in the current study [26,27,28]. Hagenmaier et al. [27,28] reported that mobility scores increased (worsened) during the marketing process. For example, Hagenmaier et al. [28] found that the prevalence of mobility scores > 1 increased from 7.1% after transport to 20.3% after lairage at the plant and similar changes were reported in Hagenmaier et al. [27], with comparable values in the current study as well. Although the impact of weather parameters on mobility outcomes was not explored in the Hagenmaier et al. studies [27,28], it should be noted that both studies were conducted in the summer and mean THIs reported exceeded the mean THI in the current study. Although using a different mobility scale, Boyd et al. [26] also reported incremental decreases in the percentage of cattle with normal mobility from shipment, to unloading at the slaughter facility, to movement up to the restrainer for slaughter during the summer.

Much of the previously published research that has included mobility as one of the outcomes has focused on numerous risk factors. The authors are unaware of studies that have explored the impact of weight or DOF on mobility challenges in cattle. Some studies do report bodyweights but often simply as a sample population characteristic rather than a factor for exploration. Thomson et al. [29] reported and named the Fatigued Cattle Syndrome (FCS), which is a syndrome identified in cattle at slaughter characterized by altered breathing, lameness, reluctance to move, and changes in physiological metabolites. Thomson et al. [29] also identified bodyweight as a potential risk factor for FCS. Additionally, heavy weight pigs have been associated with increased prevalence of the similar syndrome in pigs [30], the Fatigued Pig Syndrome (FPS), thus identifying weight as a factor worth further study. Although the current study did not specifically look at FCS, FCS does include mobility challenges and therefore future work could document additional signs of FCS.

Despite the fact that DOF and weight have not been explored and sometimes not even reported in other studies, they were shown to affect mobility in the current study. As average live weight increased, so did the percentage of mobility scores ≥ 2 and similarly as DOF increased, so did the prevalence of mobility scores ≥ 2. One of the main consequences of the reduced slaughter capacity due to COVID-19 was increased DOF and resulting weights as cattle remained in the feedlot for an extended period of time. Thus, these two factors were of interest in this study particularly. As noted on 1 July 2020, feedlots with capacity of 1000 or more head had 252% greater COF > 180 days compared to July 2019 [11]. Although there is no historical DOF data from the current study facility, when comparing DOF reported during the study period with Elanco’s benchmarking data for the similar region in prior years, the observed change in DOF is approximately 45 days. Keeping all other impacting factors identified in this study constant, adding even 30 DOF would increase the prevalence of mobility scores ≥ 2 by 9.28%. Despite the fact that this sample population of cattle experienced increased DOF, the average live weight remained stagnant. Producers implemented changes in their feeding programs to successfully minimize gain during this COVID-recovery period. Additionally, producers and processors worked together to prioritize slaughter of heavier weight cattle to avoid potential negative impacts on welfare, such as increased mobility challenges, difficulty of handling and increased bruising due to increased size [31]. The data in this study suggests that if the average weight of cattle increased even 13.6 kgs with all other factors remaining constant, the percentage of cattle with mobility scores ≥ 2 would have increased by 6.08%. Perhaps the increased collaborative efforts and communication between producers and processors during this critical time period helped reduce potential adverse problems with cattle mobility. To the authors’ knowledge, this study is the first to quantify the impact of a significant increase in DOF has on mobility in cattle and will provide critical knowledge for future events that may have similar downstream effects.

There is also limited information regarding the impact of sex class on mobility scores. In the current study it was found that sex class impacted both the percentage of 2s and 3s. Comparing sex class alone, being a heifer increased the percentage of mobility scores ≥ 2 by 43.86%. Contrary to what was found in the current study, Lee et al. [18] found a greater prevalence of mobility scores > 1 in steers compared to heifers and mixed sex class lots; however, this difference was not statistically significant. Future research should continue to assess the impacts of sex class on mobility and its interaction with other important factors as the current literature has reported varying results.

Weather parameters, such as temperature and THI, have been identified as risk factors associated with increased mobility challenges in multiple studies [17,32]. A study conducted by Gonzalez et al. [17] found that cattle were more likely to become non-ambulatory or lame when they were transported in temperatures greater than 30 °C. Conversely, Lee et al. [18] did not identify an effect of THI or temperature on mobility score but did indicate that many of their observations were made earlier in the day when maximum daily temperatures were not necessarily experienced. The average temperature during the current study period was 25 °C but there was a subpopulation of lots that were scored when the THI was characterized as Severe Stress (i.e., 79 ≤ THI). The THI was developed to account for the risk posed by high humidity coupled with high temperature [20], as cattle are less able to utilize evaporative cooling in high humidity, high temperature conditions [32]. In the current study, as THI increased, the percentage of cattle scoring 2 or 3 using the NAMI mobility scoring system also increased. If no other impacting factors were altered, a THI increase from No Stress to Mild Stress would increase the relative risk of mobility scores ≥ 2 by 45.76%. By comparison, increasing stress category from Mild Stress to Severe Stress would increase the relative risk of mobility scores ≥ 2 by 125.2%. This data emphasizes the impact that weather can have on mobility in cattle at the slaughter plant.

Although the THI did impact mobility outcomes in the current study, the resulting mobility scores were perhaps less severe than anticipated due to the environmental conditions that were experienced during the study period. Specifically, the prevalence of cattle with a mobility score of 3 remained relatively consistent when compared with previous years. The average maximum daily temperatures and average mean daily temperatures in July through October at the study location were greater in 2019 than in 2020. For example, the average mean temperature in September 2019 was 7.1 °C greater than in September 2020 [33]; a difference of this size could influence heat stress risk [20]. Overall, the lower temperatures and fewer extreme heat events of 2020 were a great benefit to the cattle industry during the study period as the potential for severe mobility challenges due to weather was minimized; considering the increases seen in other significant factors like DOF and weight, the mild weather was of vital importance.

Another mobility risk factor identified in this study in addition to previous studies [17,27] was the distance traveled to the slaughter facility. Prior research in Canada found that the likelihood of cattle becoming non-ambulatory or lame increased sharply after animals spent over 30 h on a truck [17]. Cattle in the present study were transported an average of 172 km, less than the average transport distance of 218.5 km (2.7 h) reported in the 2016 NBQA [16]. Although not the case in the present study, distance traveled for other cattle in the United States may have been increased due to plant closures as a result of COVID-19. In the pork industry, longer transport distances to plants in operation with open capacity was reported during the COVID-19 pandemic [12].

Previously published research has identified cattle handling as another mobility risk factor [27,28]. Aggressive cattle handling practices, especially when compounded by other factors like long haul distances, increase the risk of mobility problems on arrival at processing plants [34]. Stressful cattle handling may contribute to FCS, with some clinical signs of the syndrome including a stiff gait and reluctance to move [34]. Handling practices during loading and unloading in particular may have an impact on mobility scores at processing plants, with low-stress handling leading to better mobility scores for fed cattle [27], but additional research is needed to explore this further.

Beginning in 2019, many processors in the United States began to require BQA certification of the producers that supply cattle to their facilities and Beef Quality Assurance Transport certification for the transporters who haul cattle to their facilities as a step to help ensure low-stress handling of cattle particularly during the marketing process [35,36]. As the COVID-19 recovery period presented an increase in cattle mobility challenges due to the increased risk factors like longer DOF and greater body weights, some packers placed even more emphasis on low-stress handling to manage mobility challenges (personal communication, L.A.). The plant in the current study implemented bi-weekly conference calls to discuss performance on internal animal handling audits. When groups of cattle were difficult to move from the lairage pen to the drive alley, which was observed by plant employees during the study period, plant management enlisted the help of extra handlers and modified animal handling protocols (i.e., moved smaller groups) to ensure both regulatory and company requirements and standards for humane handling were met. Internal plant data suggests that average electric prod scores (i.e., the percentage of animals prodded during an audit) for 2020 were comparable or less than average prod scores for 2018 and 2019, indicating that the attention the plant gave to maintaining their standards of welfare during this difficult period was effective. This proactive approach to managing mobility challenges likely also played a role in minimizing the increase in severe mobility challenges. Low-stress handling has continually been shown to be an effective best management practice to reduce mobility issues during the marketing process.

## 5. Conclusions

As anticipated, due to some of the production consequences of the COVID-19 recovery period, the prevalence of non-normal mobility scores were numerically higher during the study period relative to historical data for both the facility and the industry. Although many factors may have contributed to the multifactorial nature of cattle mobility, this study identified weight, DOF, THI, sex class, and distance traveled to the plant as factors affecting mobility and many of these were impacted by the repercussions of the pandemic. The relatively mild seasonal weather at the study location during the study months was a benefit to cattle welfare as THI greatly influenced non-normal mobility risk in this study. Despite the challenges that cattle producers and processors faced during the COVID-19 recovery period, efforts to minimize the negative effects on cattle mobility were effective. The emphasis that the study’s slaughter facility and the cattle feeding industry have put on good stockmanship and low stress animal handling was critical during this recovery period. Understanding how factors such as weather, performance, and transport distance influence mobility will help producers and processors implement preventative measures to ensure cattle welfare in the future.

## Figures and Tables

**Table 1:** The North American Meat Institute (NAMI) Mobility Scoring System for scoring cattle mobility in finished cattle [19].

Mobility Score	Definition
1	Normal, walks easily, no apparent lameness, no change in gait
2	Exhibits minor stiffness, shortness of stride, slight limp, keeps up with normal cattle
3	Exhibits obvious stiffness, difficulty taking steps, obvious limp, obvious discomfort, lags behind normal cattle
4	Extremely reluctant to move even when encouraged by a handler; statue-like

**Table 2:** Description of lot level characteristics measured as potential risk factors associated with fed cattle mobility (*N* = 158 lots representing 15,388 head of cattle).

Parameter	Mean	SD
Average lot weight ^1^, kg	633.7	44.5
Humidity ^2^, %	46	26
Temperature ^2^, C	25	6.7
Temperature Humidity Index (THI) ^3^	70	8
Days on feed	206	50
Distance traveled (estimated) ^4^, km	172	164

^1^ Average live weight was determined by calculating the difference between full and empty livestock trailer weights for each load within the lot and then dividing this sum by the total number of cattle on all trailers within the lot. The value reported represents the average, weighted by head count within each lot. ^2^ Temperature and humidity were recorded using an online platform (AccuWeather, Inc., State College, PA, USA). ^3^ The THI was estimated using the recorded temperature and humidity values in the following equation: 0.8 × T + H × (T − 14.4) + 46.4 where T is temperature (C) and H is humidity [20]. ^4^ An estimate of distance traveled to the plant was determined using Google Maps (Google LLC, Mountain View, CA, USA) to calculate the distance from the plant zip code to the producer zip code. If multiple options were provided by Google Maps, the shortest route was selected.

**Table 3:** Days on feed (DOF) from the current study and historical data from an industry benchmarking program. Historical data presented represents information collected from August through October for the years 2016 through 2019.

	Days on Feed	
Sample Population	Mean	SD
Current Study ^1^	206.0	58.7
Elanco’s Benchmark Program, 2016–2019, Central Plains Region ^2^	160.1	35.5
Elanco’s Benchmark Program, 2016–2019, All US Participants ^3^	179.2	46.9

^1^ Data from 158 lots representing 15,388 head of cattle. ^2^ The Central Plains region includes Kansas and a portion of northeastern Colorado and represents data from 2,280,270 head of cattle. ^3^ This data represents all United States feedyard participants (primarily including TX, KS, NE, CO, IA, ID, WA, OR, and OK) and includes 8,291,090 head of cattle.

**Table 4:** Mean mobility scores and live weight of fed cattle from the current study and historical data from an industry benchmarking program.

	Mobility Scores ^1^, %								
Sample Population	1	IR ^2^	2	IR	3	IR	4	IR	Live Weight, kg
Current Study ^3^	74.55	66.67–87.15	24.30	12.85–30.85	1.14	0–1.55	0.01	0–0	633.7 ^5^
Elanco’s Cattle Mobility Assessment Database, Current Study Facility ^4^	96.19	95.33–98.08	2.41	1.42–2.99	1.38	0–1.82	0.010	0–0	632.8
Elanco’s Cattle Mobility Assessment Database, All Facilities ^5^	89.32	87.04–97.37	9.54	1.77–12.2	1.08	0–1.2	0.048	0–0	631.4

^1^ Mobility scores were defined as: 1 = normal, walks easily, no apparent lameness, no change in gait; 2 = exhibits minor stiffness, shortness of stride, slight limp, keeps up with normal cattle; 3 = exhibits obvious stiffness, difficulty taking steps, obvious limp, obvious discomfort, lags behind normal cattle; and 4 = extremely reluctant to move even when encouraged by a handler; statue-like [19]. ^2^ IR represents the interquartile range. ^3^ Data from 158 lots representing 15,388 head of cattle. ^4^ Historical data from Elanco’s Cattle Mobility Assessment database for the current study facility from August, September and October in years 2016–2019 representing 314,935 head of beef cattle (no Holsteins or cows). ^5^ Historical data from Elanco’s Cattle Mobility Assessment database representing 15 plants and 2,430,847 beef cattle (no Holsteins or cows) from August, September, and October in 2016–2019. Average live weight was determined by calculating the difference between full and empty livestock trailer weights for each load within the lot and then dividing this sum by the total number of cattle on all trailers within the lot. The value reported represents the average, weighted by head count within each lot.

**Table 5:** Final logistic regression models of the lot (*n* = 158) level risk factors associated with mobility scores ^1^ ≥ 2. and ≥ 3 in fed cattle.

		Final Logistic Regression Model Effects *p*-Values	
Parameter	*n*	Mobility Score ≥ 2	Mobility Score ≥ 3
THI Category ^2^		<0.0001	0.0114
No Stress ^3^	75		
Mild Stress ^3^	70		
Severe Stress ^3^	13		
Distance Category^4^		0.0007	0.0117
<97 km	52		
between 97 and 193 km	73		
≥193 km	33		
Sex Class		0.0101	-
Steer	72		
Heifer	74		
Mixed	12		
Days on feed	155	0.0003	-
Average Lot Weight ^5^	158	0.0020	-

^1^ Mobility scores were defined as: 1 = normal, walks easily, no apparent lameness, no change in gait; 2 = exhibits minor stiffness, shortness of stride, slight limp, keeps up with normal cattle; 3 = exhibits obvious stiffness, difficulty taking steps, obvious limp, obvious discomfort, lags behind normal cattle; and 4 = extremely reluctant to move even when encouraged by a handler; statue-like [19]. ^2^ The THI was estimated using the recorded temperature and humidity values in the following equation: 0.8 × T + H × (T − 14.4) + 46.4 where T is temperature (C) and H is humidity [20]. ^3^ The THI stress categories indicating heat load risk are defined as: No Stress = THI < 72; Mild Stress = 72 ≤ THI < 79; Severe Stress = 79 ≤ THI < 89. ^4^ An estimate of distance traveled to the plant was determined using Google Maps (Google LLC, Mountain View, CA, USA) to calculate the distance from the plant zip code to the producer zip code. If multiple options were provided by Google Maps, the shortest route was selected. ^5^ Average live weight was determined by calculating the difference between full and empty livestock trailer weights for each load within the lot and then dividing this sum by the total number of cattle on all trailers within the lot.

**Table 6:** The modeled relative risks associated with significant regression model components impacting the prevalence of non-normal mobility scores ^1^.

	Modeled Relative Risk	
	Mobility Scores	
Factor	% ≥2′s	% ≥3′s
THI Stress Category ^2^		
Mild Stress vs. No Stress	+45.76%	+63.30%
Severe Stress vs. Mild Stress	+125.20%	+263.58%
Distance Category ^3^		
“between 97 and 193 km” vs. “<97 km”	+44.95%	+197.43%
“≥193 km” vs. “<97 km”	+17.18%	+146.02%
Sex Class		
Heifer vs. Steer	+43.86%	-
Mixed vs. Steer	-2.48%	-
Days on Feed (DOF)		
Add 10 DOF	+3.00%	-
Add 30 DOF	+9.28%	-
Average Lot Weight ^4^		
Add 13.6 kg	+6.08%	-
Add 45.4 kg	+21.76%	-

^1^ Mobility scores were defined as: 1 = normal, walks easily, no apparent lameness, no change in gait; 2 = exhibits minor stiffness, shortness of stride, slight limp, keeps up with normal cattle; 3 = exhibits obvious stiffness, difficulty taking steps, obvious limp, obvious discomfort, lags behind normal cattle; and 4 = extremely reluctant to move even when encouraged by a handler; statue-like [19]. ^2^ The THI was estimated using the recorded temperature and humidity values in the following equation: 0.8 × T + H × (T − 14.4) + 46.4 where T is temperature (C) and H is humidity [20]. The THI stress categories indicating heat load risk are defined as: No Stress = THI < 72; Mild Stress = 72 ≤ THI < 79; Severe Stress = 79 ≤ THI < 89. ^3^ An estimate of distance traveled to the plant was determined using Google Maps (Google LLC, Mountain View, CA, USA) to calculate the distance from the plant zip code to the producer zip code. If multiple options were provided by Google Maps, the shortest route was selected. ^4^ Average live weight was determined by calculating the difference between full and empty livestock trailer weights for each load within the lot and then dividing this sum by the total number of cattle on all trailers within the lot.

## Data Availability

Restrictions apply to the availability of these data. Please contact the corresponding author with enquires.
